# An integrated, multiregional, and multimodal cell atlas of Alzheimer’s disease

**DOI:** 10.1002/alz70861_108223

**Published:** 2025-12-23

**Authors:** Kyle J Travaglini, Yi Ding, Mariano I Gabitto, Nadia Postupna, Joseph T Mahoney, Eitan S Kaplan, Jeff Goldy, Anish Bhaswanth Chakka, Andreas Tjaernberg, Omar Kana, Anamika Agrawal, Ming Xiao, Tejas S Bajwa, Victoria M Rachleff, Nick Dee, Erica J Melief, Jeanelle Ariza, Brian Long, Emily Gelfand, Samuel T Barlow, Darren Bertagnolli, Jazmin Campos, John S Campos, Thanh Cardenas, Tamara Casper, Rushil Chakrabarty, Michael Clark, Nasmil J. Valera Cuevas, Martin Darvas, Song‐Lin Ding, Christine L Mac Donald, Rebecca Ferrer, Jessica Gloe, Junitta Guzman, Windy Ho, Lisa M Keene, Sarah Khawand, Mitchell D Kilgore, Amanda Keen, Mckaila Leytze, Zoe Maltzer, Naomi Martin, Rachel McCue, Delissa McMillen, Gonzalo Mena, Emma Meyerdierks, Kelly P Meyers, Mark Montine, Beagan Nguy, Nicholas Peña, Amber L Nolan, Julie K Nyhus, Paul A Olsen, Maiya Pacleb, Chelsea M Pagan, Trangthanh Pham, Christine Rimorin, Giuseppe A Saldi, Sarah Walling‐Bell, Aimee M Schantz, Nadiya V Shapovalova, Staci A Sorensen, Susan M Sunkin, Michael Tieu, Amy Torkelson, Jack Waters, Angela M Wilson, David R Haynor, Nicole M Gatto, Suman Jayadev, Shubhabrata Mukherjee, Paul K Crane, Caitlin S Latimer, Boaz P Levi, Kimberly A Smith, Thomas J Grabowski, Eric B Larson, Jennie L Close, Rebecca D Hodge, Jeremy A Miller, Michael J Hawrylycz, C. Dirk Keene, Ed S Lein

**Affiliations:** ^1^ Allen Institute for Brain Science, Seattle, WA USA; ^2^ University of Washington, Seattle, WA USA; ^3^ Carnegie Melon University, Pittsburgh, PA USA; ^4^ Kaiser Permanente Washington Health Research Institute, Seattle, WA USA; ^5^ University of Washington, School of Medicine, Seattle, WA USA; ^6^ Kaiser Permanente Washington, Seattle, WA USA

## Abstract

**Background:**

Alzheimer’s disease (AD) is clinically characterized by a progressive cognitive decline associated with stereotyped accumulation of amyloid‐beta (Aβ) plaques and hyperphosphorylated Tau (pTau) tangles across brain regions. While histopathology has revealed patterns of regional involvement, the molecular and cellular events that accompany and potentially drive this progression remain incompletely understood.

**Method:**

We applied single nucleus RNA sequencing (RNAseq), ATAC‐seq, and Multiome profiling to over 7 million high‐quality nuclei from 10 brain regions—spanning medial and lateral entorhinal cortices, hippocampus, multiple temporal and frontal cortical areas, and primary visual cortex—sampled from the same cohort of 84 aged human donors across the AD spectrum (3 regions from all donors, 7 from those without severe co‐morbidities). We predicted the cell‐type for each nucleus by mapping to an expanded BRAIN initiative cell‐type taxonomy, which included AD‐associated non‐neuronal states and ∼70 brain region‐specific neuronal types. These datasets were paired with regional quantitative measurements of Aβ (6e10), pTau (AT8), and other protein pathologies, as well as cellular stains for neurons, microglia, and astrocytes.

**Result:**

We inferred two distinct protein pathology accumulation patterns across brain regions: neocortical areas accumulated Aβ prior to pTau, whereas hippocampus and entorhinal cortex had early and, in some cases, substantial pTau burden independent of Aβ. Among neocortical regions, accumulation of AT8 beyond the temporal medial lobe strongly associated with dementia. In analyzing cell‐type abundance differences associated with higher levels of pTau pathology, we identified shared motifs of selective neuronal loss consistent with our previous observations from the middle temporal gyrus. These included loss of L2/3 and L5 intratelencephalic excitatory neurons and several types of inhibitory interneurons (e.g., Vip, Sst, Pvalb). These same vulnerable inhibitory types were also reduced in hippocampal and entorhinal regions, when present, and we observed parallel increases in astrocyte, microglial, and oligodendrocyte precursor cells. Additionally, we observed a decrease in specific, regionally specialized neuron subtypes in the hippocampus, entorhinal cortex, and visual cortex.

**Conclusion:**

Our multimodal, multi‐region single‐cell atlas reveals common and region‐specific patterns of cellular vulnerability in AD. These cell‐types, particularly those commonly affected in distinct neural circuits, could serve as candidate therapeutic targets and biomarkers.

